# Satisfactory clinical outcome of operative and non-operative treatment of avulsion fracture of the hamstring origin with treatment selection based on extent of displacement: a systematic review

**DOI:** 10.1007/s00167-020-06222-y

**Published:** 2020-08-18

**Authors:** Hijleke J. A. Nauta, Anne D. van der Made, Johannes L. Tol, Gustaaf Reurink, Gino M. Kerkhoffs

**Affiliations:** 1 Department of Orthopaedic Surgery, Amsterdam UMC, University of Amsterdam, Amsterdam Movement Sciences, Meibergdreef 9, 1105AZ Amsterdam, the Netherlands; 2 Academic Center for Evidence-Based Sports Medicine (ACES), Amsterdam UMC, Amsterdam, the Netherlands; 3 Amsterdam Collaboration for Health and Safety in Sports (ACHSS), AMC/VUmc IOC Research Center, Amsterdam, the Netherlands; 4 Aspetar, Orthopaedic and Sports Medicine Hospital, Doha, Qatar

**Keywords:** Apophysis, Apophyseal, Ischium, Ischial tuberosity, Biceps femoris, Semitendinosus, Semimembranosus, Treatment, Surgery, Surgical, Conservative, Nonsurgical, Bony avulsion, Avulsion fracture, Ischial apophysis, Hamstrings, Operative, Intervention, Non-operative, Rehabilitation

## Abstract

**Purpose:**

To compare outcome of operative and non-operative treatment of avulsion fractures of the hamstring origin, with minor (< 1.5 cm) and major (≥ 1.5 cm) displacement, and early (≤ 4 weeks) and delayed (> 4 weeks) surgery.

**Methods:**

A systematic literature search was performed using PubMed, Cochrane, Embase, CINAHL and SPORTDiscus. A quality assessment was performed using the Physiotherapy Evidence Database (PEDro) scale.

**Results:**

Eight studies with 90 patients (mean age: 16 years) were included. All studies had low methodological quality (PEDro score ≤ 5). Operative treatment yielded a return to preinjury activity rate (RTPA) of 87% (95% CI: 68–95), return to sports (RTS) rate of 100% (95% CI: 82–100), Harris hip score (HHS) of 99 (range 96–100) and a University of California Los Angeles activity scale (UCLA) score of 100%. Non-operative treatment yielded a RTPA rate of 100% (95% CI:68–100), RTS rate of 86% (95% CI: 69–94), HHS score of 99 (range 96–100), and non-union rate of 18% (95% CI: 9–34). All patients with minor displacement were treated non-operatively (RTPA: 100% [95% CI: 21–100], RTS: 100% [95% CI: 51–100]). For major displacement, operative treatment led to RTPA and RTS rates of 86% (95% CI: 65–95) and 100% (95% CI: 84–100), and 0% (0/1, 95% CI: 0–79) and 100% (95% CI: 51–100) for non-operative treatment. Early surgery yielded RTPA and RTS rates of 100% (95% CI: 34–100 & 57–100) compared to 100 (95% CI: 72–100) and 90% (95% CI: 60–98) for delayed repair.

**Conclusion:**

All included studies have high risk of bias. There is only low level of evidence with a limited number of included patients to compare outcome of operative and non-operative treatment. Overall outcome was satisfactory. There is a treatment selection phenomenon based on displacement, with acceptable outcome in both groups. There is insufficient data to draw conclusions regarding timing of surgery.

**Level of evidence:**

IV

**Electronic supplementary material:**

The online version of this article (10.1007/s00167-020-06222-y) contains supplementary material, which is available to authorized users.

## Introduction

An avulsion fracture involves the forceful detachment of a bony fragment at the insertion of a tendon or ligament [10, 16]. At the proximal hamstring attachment, this type of injury generally involves the ischial apophysis. The apophysis is a secondary ossification center and represents the weakest element of the muscle–tendon-bone attachment in skeletally immature patients [4] due to incomplete closure. While full closure does not occur until late adolescence [7, 10, 11, 18] avulsion fractures of the pelvis most frequently occur among younger athletes; 95% of these injuries occur between 13 and 17 years of age [1, 4, 8, 18].

Two recent systematic reviews [3, 4] on treatment outcome of pelvic avulsion fractures showed an overall higher ‘excellent outcome’ rate and return to sports (RTS) rate compared to non-operative treatment. Both reviews concluded that, especially in patients with high functional demand and with a fragment displacement greater than 1.5 cm, operative repair should be considered [3, 4].

The main limitation of these two recent reviews is that they did not distinguish between different avulsion fracture sites and thus did not report separate data for avulsion fractures of the hamstring origin.

The main aim of this review was to evaluate clinical and radiological outcome of operative and non-operative treatment for proximal avulsion fractures of the hamstring origin. Secondary aims were to assess outcome for minor (< 1.5 cm) and major (≥ 1.5 cm) fragment displacement, as well as early (≤ 4 weeks) and delayed (> 4 weeks) surgery. Our hypothesis was that operative treatment yields superior clinical outcome, especially in avulsion fractures with displacement ≥1.5 cm. We expected early surgery to yield better clinical outcome than delayed surgery.

## Materials and methods

### Search strategy

A literature search using PubMed, Cochrane, Embase, CINAHL and SPORTDiscus was performed to identify potentially eligible articles up to 12 December 2019. There was no restriction on publication date. The search strategy per database can be found in the supplementary appendix.

### Study selection

Selection of potentially eligible studies was performed using web app Rayyan [12] (QCRI, Doha, Qatar). Duplicates were removed. Using the eligibility criteria in Fact box 1, two reviewers (HJAN & ADM) independently assessed article eligibility based on title and abstract, followed by assessment of full-texts. If there was any doubt regarding eligibility based on screening of title and abstract, the study was moved forward to full-text screening. If no consensus was reached after assessing the full-text, a third reviewer was available. Citation tracking of included full-texts was performed after screening of full-texts. For any full-text that was not available, authors were contacted by email.
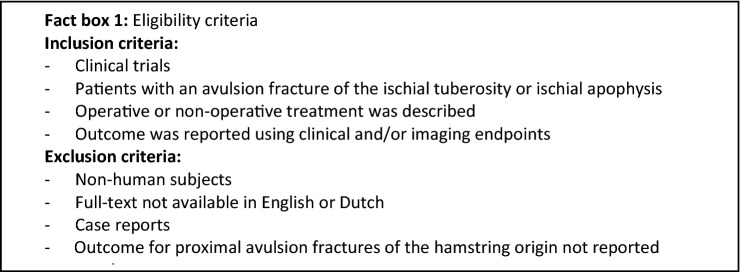


### Data extraction and synthesis

Relevant data was extracted independently by two authors (HJAN & ADM) using a standardised data extraction form. This form included study design, study population, duration of follow-up, avulsion fragment displacement, treatment modality, complications, and outcome measures. In case of uncertainties, a third author was consulted. Outcome measures that were used in multiple included studies were pooled.

### Risk of bias assessment

To assess the risk of bias of the included studies, The PEDro Scale [17] was used by us. Assessment was done independently by two reviewers (HJAN & ADM). In case consensus was not reached, assessment by a third reviewer was decisive. The PEDro scale uses eleven items (Fact box 2) to score the methodological quality of the included studies. Each appraisal item could be scored with ‘no’ or ‘yes’. The first item of the scale relates to external validity and is not included in the final score. This means that the final score (0–10) is calculated using items 2–11. A PEDro score of ≥ 6 indicates a low risk of bias (i.e., high-quality) study. A score of ≤ 5 indicates a high risk of bias study (i.e., low-quality study).
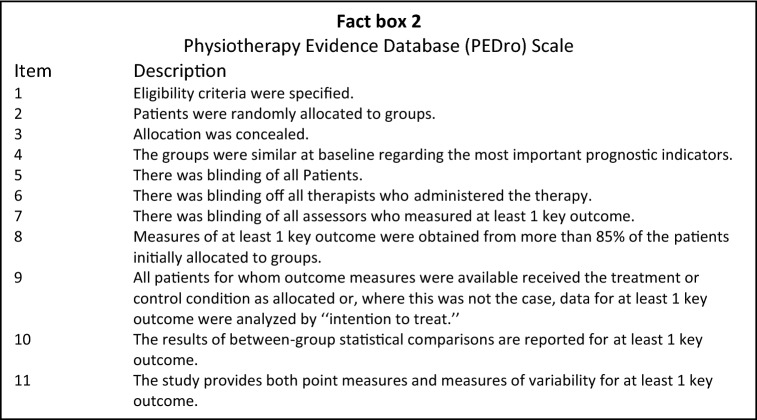


### Evidence synthesis

Data from studies with low risk of bias will be pooled if available from multiple studies. If no studies with low risk of bias are available, data from studies with high risk of bias will be pooled. If data cannot be pooled, a best evidence synthesis will be done.

For pooled outcomes given as a proportion, 95% confidence intervals (95% CI, binomial proportion) using a Wilson score interval were calculated by us. If 95% confidence intervals overlapped indicating absence of statistically significant differences, no further statistical testing of between-group differences was performed.

## Results

**Fig. 1:**
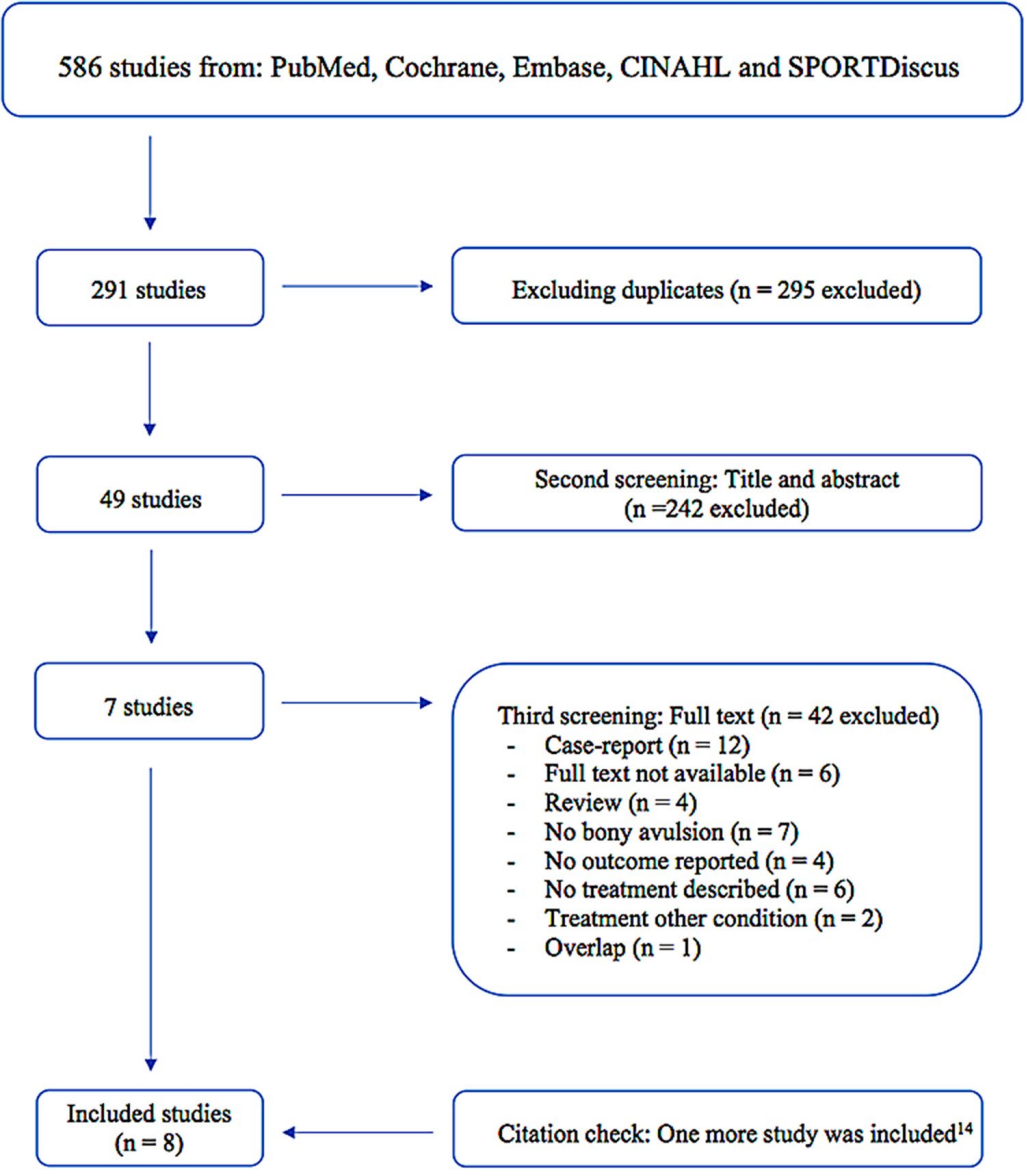
Flowchart of study selection

The literature search identified 586 potentially eligible study. After study selection, 8 studies were included [2, 5, 6, 9, 10, 13–15] (Fig. 1).

### Study characteristics

The eight included studies were all case series (Table 1), with a total of 90 patients and a weighted mean age of 16 years (range in study means 14–19). Gender was reported for 79 patients (13 females and 66 males). Operative treatment was chosen in 27 patients and 63 patients were treated non-operatively.

**Table 1:** Study characteristics

Study	Study design (follow-up in months)	Patients, sex, weighted mean age	Timing of surgery (≤ 4 or > 4 weeks)	Intervention	Displacement (< 1.5/ ≥ 1.5 cm)	Outcome measure	Outcome non-operative	Outcome operative	Outcome secondary operative
Shyamalan and Bircher [14]	Case series (6–48)	*N* = 4 (out of 8) F = 0, M = 4 A = 15 (11–22)	Early: 0 Delayed: 4	NO: 0 O: 4	0/3 ≥ (1 NR)	RTPA		100% (4/4)	
Kujala et al. [9]	Case series (≥ 3)	*N* = 21 F = 3, M = 18 A = 19 (range 13–41)	Early: 0 Delayed: 7	NO: 21 O: 0 SO: 7	(20 NR) / 1 (20 NR)	SO rate	33% (7/21)		
						Outcome grading†	50% Excellent (7/14) 21% Good (3/14) 29% Moderate (4/14)		29% Excellent (2/7) 57% Good (4/7) 14% Moderate (1/7)
						RTS	71% (10/14)		86% (6/7)
Sinikumpu et al. [15]	Case series (≥ 12)	*N* = 11 (out of 32) F/M = NR A = NR	NR	NO: 0 O: 11	0/11	Outcome grading†		73% Good (8/11) 27% Moderate (3/11)	
						RTS		100% (11/11)	
						RTPA		73% (8/11)	
Ferlic et al. [5]	Retrospective case series (≥ 24)	*N* = 13 F = 1, M = 12 A = 15 (range 13- 16)	Early: 4 Delayed: 1	NO: 9 O: 4 SO: 1	4/9	SO rate	11% (1/9)°		
						Outcome grading†	75% Excellent (6/8) 25% Good (2/8)	100% Excellent (4/4)	100% Good (1/1)
						RTS	100% (8/8)	100% (4/4)	100% (1/1)
						HHS	Mean 99 (range 96–100)	Mean 99 (range 96–100)	
						Non-union	25% (2/8)		
Biedert et al. [2]	Case series (12–24)	*N* = 3 F = 0, M = 3 A = 14 (range 13- 15)	Early: 1 Delayed: 2	NO: 0 O: 3	0/3	RTS		100% (3/3)	
						RTPA		100% (3/3)	
						TAS		Mean 98 (range 95–100)	
						UCLA		100% regularly participate in impact sports	
Schuett et al. [13]	Retrospective case series (9.6)	*N* = 25 (out of 225) F = 7, M = 18 A = 14 (NR)	N/A	NO: 25 O: 0	NR	Non-union	16% (4/25)		
Gidwani and Bircher [6]	Retrospective case series (NR)	*N* = 7 (out of 12) F = 1, M = 6 A = 18 (range 9–32)	Early: 1 Delayed: 4	NO: 2 O: 5	1/5 (1 NR)	Outcome grading†	50% Good (1/2) 50% Satisfactory (1/2)	100% Good (5/5)	
						RTPA	100% (2/2)	100% (5/5)	
Metzmaker and Pappas [10]	Case series (≥ 18)	*N* = 6 (out of 27) F = 1, M = 5 A = 15 (range 13- 17)	N/A	NO: 6 O: 0	NR	Outcome grading†	83% Excellent (5/6) 17% Good (1/6)		
						RTS	100% (6/6)		
						RTPA	100% (6/6)		

*RTS* return to sports, *RTPA* return to pre-injury activity level, *HHS* Harris Hip score, *UCLA* University of California Los Angeles activity scale, *TAS* Tegner Activity Scale, *NR* not reported, *N* sample size, *F/M* female/male, *A* mean age (years), *No* non-operative, *O* operative, *SO* secondary operative. °No outcome reported on non-operative treatment prior to secondary operation. †For grade definitions per study, see Table 3

### Risk of bias assessment

The outcome of the risk of bias assessment is shown in Table 2. All assessed articles scored ≤ 5 and were considered to have high risk of bias, mainly due to the absence of blinding, randomization and control groups.

**Table 2:** Risk of bias assessment of the included Studies using the PEDro Scale [17]

Study	1	2	3	4	5	6	7	8	9	10	11	Total score
Shyamalan and Bircher [14]	Yes	No	No	No	No	No	No	Yes	Yes	No	No	2/10
Kujala et al. [9]	Yes	No	No	No	No	No	No	Yes	Yes	No	No	2/10
Sinikumpu et al. [15]	Yes	No	No	No	No	No	No	Yes	Yes	Yes	No	3/10
Ferlic et al. [5]	Yes	No	No	No	No	No	No	Yes	Yes	No	Yes	3/10
Biedert et al. [2]	Yes	No	No	No	No	No	No	Yes	Yes	No	No	2/10
Schuett et al. [13]	Yes	No	No	No	No	No	No	No	Yes	Yes	No	2/10
Gidwani and Bircher [6]	No	No	No	No	No	No	No	Yes	Yes	No	No	2/10
Metzmaker and Pappas [10]	Yes	No	No	No	No	No	No	Yes	Yes	No	No	2/10

### Outcome measures

Outcome measures used in the included studies were return to sports rate (RTS), return to preinjury activity rate (RTPA), outcome grading based on a 4-point outcome grading system shown in Table 3 (poor, moderate, good, excellent) [2, 5, 6, 9, 10, 13–15], Harris hip score (HHS) [5], rate of non-union [5, 13], Tegner Activity Scale (TAS), and University of California Los Angeles (UCLA) activity scale [2].

**Table 3:** Outcome grading system as reported by the different studies

Study	Excellent	Good	Moderate/fair	Poor
Kujala et al. [9]	All sports possible without limitations and pain	Some pain in strenuous sports	Patient had to stop competitive sports, but no severe limitations in normal life	
Sinikumpu et al. [15]	Ability to return to preinjury sports level		Inability to return to preinjury activity level, because of significant pain and discomfort during such activity with only minor symptoms during daily activities	Discomfort in activities of daily living
Ferlic et al. [5]	Sports activities possible without restrictions	Occasional pain during sports and/or daily routine	Impossibility to perform sports activities	
Metzmaker and Pappas [10]	Full return to preinjury status within 4 months	Return to preinjury sport but continued to report an intermittent local aching sensation	Able to return to competition, but nor at the previous level	Unable to return to competition at any level due to weakness or pain

### Outcome following operative and non-operative treatment

Operative treatment was chosen in 27 patients (6 studies) and non-operative treatment in 63 patients (5 studies). Outcome per group is presented in Table 4. Fourteen patients that were initially treated non-operatively proceeded to undergo secondary operative treatment. Excluding these cases, operative repair led to good-to-excellent outcome in 85%, compared to 83% after non-operative treatment. An RTPA rate of 87% (20/23) and RTS rate of 100% (18/18) in the operative group, compared to 100% (8/8) and 86% (24/28) in the non-operative group, was calculated. Non-unions were only reported in the non-operative group, and occurred in 18% (6/33). There was no difference between the groups in terms of mean HHS (both 99, range 96–100). The UCLA score was 100%, but was only reported in three patients in the operative group.

**Table 4:** Outcomes for operative, non-operative and secondary operative treatment for avulsion fractures of the hamstring origin

	Treatment		
	Non-operative	Operative	Secondary operative
Outcome grading: good to excellent	83% (95% CI: 66–93) (25/30)	85% (95% CI: 64–95) (17/20)	88% (95% CI: 53–98) (7/8)
RTPA	100% (95% CI:68–100) (8/8)	87% (95% CI: 68–95) (20/23)	–
RTS	86% (95% CI: 69–94) (24/28)	100% (95% CI: 82–100) (18/18)	88% (95% CI: 53–98) (7/8)
HHS	Mean 99 (range 96–100)	Mean 99 (range 96–100)	
Non-union	18% (95% CI: 9–34) (6/33)	–	–
UCLA score	–	100%	–

*RTPA* return to pre-injury activity level, *RTS* return to sports, *HHS* Harris Hip score, *UCLA* University of California Los Angeles (activity scale), *NR* not reported

### Outcome of secondary operative treatment

For 8 patients that underwent secondary operative treatment outcome was reported separately, and a RTS rate of 88% was calculated. Reported causes of unsatisfying results in these patients were non-union, hamstring syndrome, pseudotumor, calcified fragments and sciatic nerve entrapment.

### Outcome of treatment and fragment displacement

Six studies (*N* = 37) reported extent of fragment displacement. Patients were divided in two groups: < 1.5 cm (*N* = 5) and ≥ 1.5 cm (*N* = 32) displacement (Table 5). All 5 patients with < 1.5 cm displacement were treated non-operatively, with a RTPA rate of 100% (1/1) and RTS rate of 100% (4/4). Thirty-two patients had a displacement of ≥ 1.5 cm from which 27 (84%) were treated operatively and 5 (16%) were treated non-operatively. The operated treatment group had RTPA and RTS rates of 86% (18/21) and 100% (20/20). In the non-operative treatment group, the RTPA and RTS rates were 0% (0/1) and 100% (4/4).

**Table 5:** Outcome of operative and non-operative treatment of avulsion fractures of the hamstring origin with minor (< 1.5 cm) and major (≥ 1.5 cm) displacement)

	Fragment displacement	
	< 1.5 cm	≥ 1.5 cm
Operative		
RTPA	–	86% (95% CI: 65–95) (18/21)
RTS	–	100% (95% CI: 84–100) (20/20)
Non-operative		
RTPA	100% (95% CI: 21–100) (1/1)	0% (95% CI: 0–79) (0/1)
RTS	100% (95% CI: 51–100) (4/4)	100% (95% CI: 51–100) (4/4)

*RTPA* Return to Pre-injury Activity level. RTS: Return to Sports

### Outcome of treatment and timing of surgery

In five studies (*N* = 24), both timing of surgery and treatment outcome were reported (Table 6) [2, 5, 6, 9, 14]. Six patients underwent early surgery (≤ 4 weeks post injury), and all (100%) returned to pre-injury activity level (2/2) and returned to sports (5/5).

**Table 6:** Outcome of early (≤ 4 weeks after injury) and delayed (> 4 weeks after injury) surgery

	Timing of surgery	
	Early (≤ 4 weeks)	Delayed (> 4 weeks)
RTPA	(2/2) 100% (95% CI: 34–100)	(10/10) 100% (95% CI: 72–100)
RTS	(5/5) 100% (95% CI: 57–100	(9/10) 90% (95% CI: 60–98)

*RTPA* return to pre-injury activity level, *RTS* return to sports

Eighteen patients underwent delayed surgery (> 4 weeks post injury), with RTPA and RTS rates of 100% (10/10) and 90% (9/10).

## Discussion

The most important finding of the present study was that only studies with high risk of bias (PEDro score ≤ 5) and a limited number of included patients were available to compare treatment outcome of operative and non-operative treatment for avulsion fractures of the hamstring origin. The clinical outcome in both groups was satisfactory with high RTPA and RTS rates. The comparison is further limited by the fact that avulsion fractures with minor (< 1.5 cm) fragment displacement were all treated non-operatively indicating a selection phenomenon. Given the low level of evidence, it remains unclear which intervention is preferred. This review serves to provide an overview of currently available literature for clinicians and has identified the gaps in current evidence for future research efforts.

Overall, both operative and non-operative treatment resulted in satisfactory outcome. The group with minor avulsion fragment displacement (< 1.5 cm) had good outcome with non-operative treatment, but no data is available to compare it to outcome of operative treatment in this group. In the group with major (≥ 1.5 cm) fragment displacement, outcome in terms of RTS and RTPA are generally acceptable. For timing of operative repair, early repair (≤ 4 weeks) resulted in RTS and RTPA rates similar to the delayed repair (> 4 weeks) group, but data is scarce.

There are no other systematic reviews that have investigated outcome of avulsion fractures of the hamstring origin in isolation. Eberbach et al. [4] pooled all pelvic avulsion fractures and reported overall success rates of 88 and 79% for operative and non-operative treatment, respectively (n.s.). In addition, RTS rates of 92 and 80% were reported (*p* = 0.03). The review of Calderazzi et al. [3] reported similar findings. Operative treatment resulted in a RTS rate of 95% compared to 90% for non-operative treatment. The authors advocated operative treatment for avulsion fractures with greater fragments and major displacement.

These results appear to be in line with our findings in avulsion fractures of the hamstring origin. For the relationship between extent of avulsion fragment displacement and treatment outcome, the same cut-off of 1.5 cm as Eberbach et al. [4] was used. Their review concluded that avulsion fractures with less than 1.5 cm displacement could be treated non-operatively. Operative treatment was recommended for avulsion fractures with more than 1.5 cm displacement. In the current systematic review, avulsion fractures with minor displacement were treated non-operatively with satisfactory outcome. The comparison with operative repair in this group could not be made due to absence of reported data. Operative and non-operative treatment of avulsion fractures with more than 1.5 cm displacement appear to result in similar outcome but it should be noted that the sample size in the non-operative group is very small. Both early and delayed surgery yielded high RTS and RTPA rates.

All included studies in this review were scored as low-quality. There was no randomization, blinding or comparison used which causes a high risk of (e.g., selection) bias. A selection phenomenon, where treatment choice was seemingly based on the extent of avulsion fragment displacement which impeded a proper comparison, was noted. Another issue introducing bias is the lack of data on the initial and apparent non-satisfactory outcome of non-operative treatment in patients that underwent secondary operative treatment. In addition, the (sub)group sizes were too small to draw firm conclusions regarding the < 1.5 cm group and timing of surgery. There was notable variation in treatment protocols used in the various studies. This is, however, the first systematic review investigating outcome of operative and non-operative treatment for proximal hamstring avulsion fractures separately.

### Implications for clinical practice and future research

In current practice, where avulsion fractures with minor (< 1.5 cm) displacement are treated non-operatively and majorly displaced (≥ 1.5 cm) avulsion fractures are predominantly treated with operative repair, overall outcome is satisfactory. Due to paucity of data and high risk of bias it remains unclear which treatment should be advised in the individual patient. On the one hand, these findings can be viewed as a confirmation of currently employed treatment decision-making based on the amount of displacement. Still, the need for comparative prospective studies and ideally randomized controlled trials is underlined to allow for a proper comparison and, by extension, development of evidence-based treatment protocols. In the meanwhile, our findings can be used to inform patients about expected outcome and guide shared-decision making.

## Conclusion

All included studies have high risk of bias. Thus, there is only low level of evidence with a limited number of included patients to compare outcome of operative and non-operative outcome for proximal avulsion fractures of the hamstring origin. Overall, satisfactory outcome was found in both groups with high RTPA and RTS rates. A selection phenomenon in which treatment is chosen based on the amount of avulsion fragment displacement, resulting in acceptable outcome in both groups, was noted. There was insufficient data to conclude whether a difference exists between early and delayed surgery.

## Electronic supplementary material

Below is the link to the electronic supplementary material.

Supplementary file1 (DOCX 13 kb)

## References

[1] Anderson SJ (2002). Lower extremity injuries in youth sports. Pediatr Clin North Am.

[2] Biedert RM (2015). Surgical management of traumatic avulsion of the ischial tuberosity in young athletes. Clin Sports Med.

[3] Calderazzi F, Nosenzo A, Galavotti C, Pogliacomi F, Ceccarelli F (2018). Apophyseal avulsion fractures of the pelvis. A review. Acta Biomed Ateneo Parmense.

[4] Eberbach H, Hohloch L, Feucht MJ, Konstantinidis L, Südkamp NP, Zwingmann J (2017). Operative versus conservative treatment of apophyseal avulsion fractures of the pelvis in the adolescents: a systematical review with meta-analysis of clinical outcome and return to sports. BMC Musculoskelet Disord.

[5] Ferlic PW, Sadoghi P, Singer G, Kraus T, Eberl R (2014). Treatment for ischial tuberosity avulsion fractures in adolescent athletes. Knee Surg Sports Traumatol Arthrosc.

[6] Gidwani S, Bircher MD (2007). Avulsion injuries of the hamstring origin - a series of 12 patients and management algorithm. Ann R Coll Surg Engl.

[7] Howard FM, Piha RJ (1965). Fractures of the apophyses in adolescent athletes. JAMA.

[8] Kocher MS, Tucker R (2006). Pediatric athlete hip disorders. Clin Sports Med.

[9] Kujala UM, Orava S, Karpakka J, Leppavuori J, Mattila K (1997). Ischial tuberosity apophysitis and avulsion among athletes. Int J Sports Med.

[10] Metzmaker JN, Pappas AM (1985). Avulsion fractures of the pelvis. Am J Sports Med.

[11] Moeller JL (2003). Pelvic and hip apophyseal avulsion injuries in young athletes. Curr Sports Med Rep.

[12] Ouzzani M, Hammady H, Fedorowicz Z, Elmagarmid A (2016). Rayyan—a web and mobile app for systematic reviews. Syst Rev.

[13] Schuett DJ, Bomar JD, Pennock AT (2015). Pelvic apophyseal avulsion fractures: a retrospective review of 228 cases. J Pediatr Orthop B.

[14] Shyamalan G, Bircher M (2010). Chronic complete proximal hamstring injury: The double-window approach for bony avulsions. Injury.

[15] Sinikumpu JJ, Hetsroni I, Schilders E, Lempainen L, Serlo W, Orava S (2018). Operative treatment of pelvic apophyseal avulsions in adolescent and young adult athletes: a follow-up study. Eur J Orthop Surg Traumatol.

[16] Tehranzadeh J (1987). The spectrum of avulsion and avulsion-like injuries of the musculoskeletal system. RadioGraphics.

[17] Verhagen AP, de Vet HCW, de Bie RA, Kessels AGH, Boers M, Bouter LM, Knipschild PG (1998). The delphi list. J Clin Epidemiol.

[18] Waite BL, Krabak BJ (2008). Examination and treatment of pediatric injuries of the hip and pelvis. Phys Med Rehabil Clin N Am.

