# Supplementary material for: Satisfactory clinical outcome of operative and non-operative treatment of avulsion fracture of the hamstring origin with treatment selection based on extent of displacement: a systematic review
Source: Knee Surg Sports Traumatol Arthrosc. 2020 Aug 18;29(6):1813–21. doi: 10.1007/s00167-020-06222-y (PMC8126544; doi:10.1007/s00167-020-06222-y)
Supplement: Supplementary file 1 — Supplementary file1 (DOCX 13 kb) [file 167_2020_6222_MOESM1_ESM.docx]

| Database | Search strategy |
| --- | --- |
| PubMed | ("Fractures, Avulsion"[Mesh] OR avulsion[tiab] OR apophyseal[tiab] OR apophysis[tiab]) AND ("Ischium"[Mesh] OR "Hamstring Muscles"[Mesh] OR ischium[tiab] OR ischial[tiab] OR hamstring*[tiab]) AND ("Surgical Procedures, Operative"[Mesh] OR "surgery" [Subheading] OR "Rehabilitation"[Mesh] OR "rehabilitation" [Subheading] OR "Conservative Treatment"[Mesh] OR surgery[tiab] OR surgical[tiab] OR operat*[tiab] OR refixat*[tiab] OR fixat*[tiab] OR reattach*[tiab] OR conservative[tiab] OR non-operat*[tiab] OR nonoperat*[tiab] OR rehabilit*[tiab] OR nonsurgical*[tiab] OR non-surgical*[tiab] OR treatment[tiab]) |
| CINAHL | ((MH "Avulsion Fractures") OR ((TI (avulsion or apophyseal or apophysis) OR AB (avulsion or apophyseal or apophysis)))) AND ((MH "Ischium") OR (MH "Hamstring Muscles") OR ((TI (ischium or ischial or hamstring*) OR AB (ischium or ischial or hamstring*)))) AND ((MH "Surgery, Operative+") OR (MH "Rehabilitation+") OR ((TI (surgery or surgical or operat* or refixat* or fixat* or reattach* or conservative or non-operat* or nonoperat* or rehabilit* or nonsurgical* or non-surgical* or treatment) OR AB (surgery or surgical or operat* or refixat* or fixat* or reattach* or conservative or non-operat* or nonoperat* or rehabilit* or nonsurgical* or non-surgical* or treatment)))) |
| SPORTdiscus | (Avulsion Fracture OR (TI (avulsion or apophyseal or apophysis) OR AB (avulsion or apophyseal or apophysis ))) AND (ischium OR hamstring OR (TI (ischium or ischial or hamstring*) OR AB (ischium or ischial or hamstring*))) AND (surgery OR rehabilitation OR conservative treatment OR (TI (surgery or surgical or operat* or refixat* or fixat* or reattach* or conservative or non-operat* or nonoperat* or rehabilit* or nonsurgical* or non-surgical* or treatment) OR AB (surgery or surgical or operat* or refixat* or fixat* or reattach* or conservative or non-operat* or nonoperat* or rehabilit* or nonsurgical* or non-surgical* or treatment))) |
| Cochrane | #1: MeSH descriptor: [Fractures, Avulsion] explode all trees. #2: (avulsion or apophyseal or apophysis):ti,ab,kw. #3: #1 or #2. #4: MeSH descriptor: [Ischium] explode all trees. #5: MeSH descriptor: [Hamstring Muscles] explode all trees. #6: (ischium or ischial or hamstring*):ti,ab,kw. #7: #4 or #5 or #6. #8: MeSH descriptor: [Surgical Procedures, Operative] explode all trees. #9: MeSH descriptor: [Rehabilitation] explode all trees. #10: MeSH descriptor: [Conservative Treatment] explode all trees. #11: (surgery or surgical or operat* or refixat* or reattach* or conservative or non-operat* or nonoperat* or rehabilit* or nonsurgical* or non-surgical* or treatment):ti,ab,kw. #12: #8 or #9 or #10 or #11. #13: #3 and #7 and #12. |
| EMBASE | ((avulsion injury/ or (avulsion or apophyseal or apophysis).ti,ab,kw.) AND (ischium/ or hamstring muscle/ or (ischium or ischial or hamstring*).ti,ab,kw.) AND (exp surgery/ or su.fs. or exp rehabilitation/ or exp conservative treatment/ or (surgery or surgical or operat* or refixat* or fixat* or reattach* or conservative or non-operat* or nonoperat* or rehabilit* or nonsurgical* or non-surgical* or treatment).ti,ab,kw.)) NOT (limit to conference abstract status) |

Search strategies per database with number of results. (CINAHL: Cumulative Index to Nursing and Allied Health Literature, Cochrane: Cochrane Central Register of Controlled Trials, EMBASE: Excerpta Medica Databank)
